# 4β-Hydroxywithanolide E from *Physalis peruviana *(golden berry) inhibits growth of human lung cancer cells through DNA damage, apoptosis and G_2_/M arrest

**DOI:** 10.1186/1471-2407-10-46

**Published:** 2010-02-18

**Authors:** Ching-Yu Yen, Chien-Chih Chiu, Fang-Rong Chang, Jeff Yi-Fu Chen, Chi-Ching Hwang, You-Cheng Hseu, Hsin-Ling Yang, Alan Yueh-Luen Lee, Ming-Tz Tsai, Zong-Lun Guo, Yu-Shan Cheng, Yin-Chang Liu, Yu-Hsuan Lan, Yu-Ching Chang, Ying-Chin Ko, Hsueh-Wei Chang, Yang-Chang Wu

**Affiliations:** 1 Department of Oral and Maxillofacial Surgery, Chi-Mei Medical Center, Tainan, Taiwan; 2 School of Dentistry, Taipei Medical University, Taipei, Taiwan; 3 Department of Biotechnology, Kaohsiung Medical University, Kaohsiung, Taiwan; 4 Graduate Institute of Natural Products, College of Pharmacy, Kaohsiung Medical University, Kaohsiung, Taiwan; 5 Department of Biochemistry, Kaohsiung Medical University, Kaohsiung, Taiwan; 6 Department of Cosmeceutics, College of Pharmacy, China Medical University, Taichung, Taiwan; 7 Institute of Nutrition, China Medical University, Taichung, Taiwan; 8 Institute of Molecular Medicine, National Tsing-Hua University, Hsinchu, Taiwan; 9 Department of Life Science, National Tsing-Hua University, Hsinchu, Taiwan; 10 School of Pharmacy, China Medical University, Taichung, Taiwan; 11 Agricultural Biotechnology Research Center, Academia Sinica, Taipei, Taiwan; 12 Division of Environmental Health and Occupational Medicine, National Health Research Institutes, Kaohsiung, Taiwan; 13 Graduate Institute of Public Health, College of Health Science, Kaohsiung Medical University, Kaohsiung, Taiwan; 14 Department of Biomedical Science and Environmental Biology, Kaohsiung Medical University, Kaohsiung, Taiwan; 15 Center of Excellence for Environmental Medicine, Kaohsiung Medical University, Kaohsiung, Taiwan

## Abstract

**Background:**

The crude extract of the fruit bearing plant, *Physalis peruviana *(golden berry), demonstrated anti-hepatoma and anti-inflammatory activities. However, the cellular mechanism involved in this process is still unknown.

**Methods:**

Herein, we isolated the main pure compound, 4β-Hydroxywithanolide (4βHWE) derived from golden berries, and investigated its antiproliferative effect on a human lung cancer cell line (H1299) using survival, cell cycle, and apoptosis analyses. An alkaline comet-nuclear extract (NE) assay was used to evaluate the DNA damage due to the drug.

**Results:**

It was shown that DNA damage was significantly induced by 1, 5, and 10 μg/mL 4βHWE for 2 h in a dose-dependent manner (*p *< 0.005). A trypan blue exclusion assay showed that the proliferation of cells was inhibited by 4βHWE in both dose- and time-dependent manners (*p *< 0.05 and 0.001 for 24 and 48 h, respectively). The half maximal inhibitory concentrations (IC_50_) of 4βHWE in H1299 cells for 24 and 48 h were 0.6 and 0.71 μg/mL, respectively, suggesting it could be a potential therapeutic agent against lung cancer. In a flow cytometric analysis, 4βHWE produced cell cycle perturbation in the form of sub-G_1 _accumulation and slight arrest at the G_2_/M phase with 1 μg/mL for 12 and 24 h, respectively. Using flow cytometric and annexin V/propidium iodide immunofluorescence double-staining techniques, these phenomena were proven to be apoptosis and complete G_2_/M arrest for H1299 cells treated with 5 μg/mL for 24 h.

**Conclusions:**

In this study, we demonstrated that golden berry-derived 4βHWE is a potential DNA-damaging and chemotherapeutic agent against lung cancer.

## Background

Lung cancer is a leading cause of cancer death in the US [1] and other countries. Many therapies for lung cancer such as radiotherapy [2], docetaxel [3], the combination of carboplatin and paclitaxel [4], and others were developed; however, drug development for lung cancer is still challenging.

Dietary chemoprevention using fruits and foods [5-7] as a practical, economical, and effective approach in reducing the risk of many cancers was reviewed [8-11]. Protection against lung cancer by a higher dietary intake of fruits and vegetables was reported in several epidemiological studies [12-15].

*Physalis peruviana *(golden berry) is a member of the Solanaceae, an edible plant family. The fruit tastes like a sweet tomato and includes high levels of vitamin C, vitamin A, and the vitamin B-complex. The fruit was demonstrated to have both anti-inflammatory and antioxidant properties [16-18]. Moreover, *P. peruviana *extracts were reported to have anti-hepatoma activity due to the induction of apoptosis in a human hepatocellular carcinoma (Hep G_2_) cell line [19].

Recently, various cytotoxic withanolides (tubocapsenolide A, tubocapsanolide A, 20-hydroxytubocapsanolide A, 23-hydroxytubocapsanolide A, tubocapsanolide F, and anomanolide B) were isolated from *Tubocapsicum anomalum *and reported to be effective against liver, lung, and breast cancer cell lines [20]. Similarly, many pure compounds such as a series of C28 steroidal lactones, physalins, and withanolides, were extracted from *P. peruviana *[21-23]; however, the bioactive compounds with anticancer activities [19] are still unclear. This raised the possibility that some withanolides from *P. peruviana *are capable of anticancer activity.

On the basis of the description of active extracts from *P. peruviana*, this plant was chosen as the basis for our investigations. Using a bioactivity-guided fractionation method, we isolated the major active compound, 4βHWE Although this was first isolated from *P. peruviana *by other researchers a long time ago [21], its possible cytotoxic role and mechanisms of action have not been addressed. Accordingly, we proposed a hypothesis that 4βHWE is one of the main potential anticancer candidates in *P. peruviana*. To test this hypothesis, we used golden berry-derived 4βHWE to investigate the anticancer potential against human lung carcinoma H1299 cells using a new toxicological technique.

## Methods

### Plant material

The plant material (10.8 kg) was collected at the Tainan District Agricultural Research and Extension Station, Taiwan, in April 2005. Before the extraction process, we deposited the original plant as a voucher specimen (standard) in the Graduate Institute of Natural Products (Kaohsiung Medical University, Kaohsiung, Taiwan) to ensure adequate collection data.

### Extraction and isolation

The aerial parts (stems and leaves) of *P. peruviana *(10.8 kg) were extracted three times with EtOH at room temperature to obtain a crude extract. The crude extract was partitioned with EtOAc and H_2_O (1:1, v/v) to give an EtOAc layer which was then partitioned with n-hexane, MeOH, and H_2_O (10:7:3, v/v/v) to give an MeOH/H_2_O layer (65.2 g). The MeOH/H_2_O layer extract was chromatographed over a silica gel column, using polarity gradient mixtures of n-hexane-EtOAc-MeOH (1:0:0, 1 L), (40:1:0, 2 L), (20:1:0, 2 L), (10:1:0, 2 L), (1:1:0, 2 L), (0:1:0, 3 L), (0:40:1, 2 L), (0:30:1, 2 L), (0:20:1, 2 L), (0:10:1, 1 L), and (0:0:1, 1 L) as eluants, and this yielded 24 fractions. The volume for each fraction was 500 ml. Fraction 15 (9154.4 mg) was subjected to Sephadex LH-20 (EtOAc:MeOH 1:4), and the subfraction (fr. 15-3-7) contained crystals which were acetone-washed to provide the main pure compound.

### Cell cultures

Cells were routinely cultured in 1× RPMI-1640 medium (Gibco, Grand Island, NY) supplemented with 10% fetal bovine serum (FBS), 100 U/mL penicillin, 100 μg/mL streptomycin, and 0.03% glutamine. All cells were kept at 37°C in a humidified atmosphere containing 5% CO_2_. A H1299 (human lung adenocarcinoma) cell line was used to test the cytotoxicity against lung cancer; an HGF-1 (human normal gingival fibroblast) [24] cell line was used as the normal control; and an NB4 (human acute promyelocytic leukemia) cell line was the nuclear extract (NE) resource for the comet-NE assay described below.

### Comet-NE assay

The protocol for the comet-NE assay was as described previously [25,26]. In brief, NEs were prepared using NB4 cells as described before [7,25,26]. The 100 μl aliquots of H1299 cell suspensions (1×10^6 ^cells/ml in phosphate-buffered saline; PBS) were mixed with equal volumes of 1.2% low-melting-point agarose (in PBS, pH 7.4) and immediately pipetted onto a slide precoated with 1.2% regular agarose (in distilled water), and the slide was placed on ice to solidify the gel. The third layer (100 μl of 1.2% low-melting agarose gel) was coated onto the solidified second gel, and then the slide was placed on ice to solidify the gel. The slides were immersed in freshly made ice-cold cell lysis solution (2.5 M NaCl, 100 mM EDTA, 10 mM Tris (pH 10), 1% N-laurylsarcosine, 1% Triton X-100, and 10% dimethyl sulfoxide (DMSO), stored at 4°C for 2 h, and rinsed with deionized water three times. A 20 μl excision mixture containing 0.6 μg NE, 50 mM Hepes-KOH (pH 7.9), 70 mM KCl, 5 mM MgCl_2_, 0.4 mM EDTA, 2 mM ATP, 40 mM phosphocreatine, and 2.5 mM creatine phosphokinase was added to each slide for NE digestion. A coverslip was applied, and the slides were then incubated at 37°C for 2 h in a humidified space. The slides were denatured in 0.3 N NaOH and 1 mM EDTA for 20 min followed by electrophoresis at 20 V and 300 mA for 25 min. After being washed with deionized water, the slides were transferred to 0.4 M Tris-HCl (pH 7.5). Then 40 μl propidium iodide (PI, 50 μg/mL) was added. For fluorescence microscopy (TE2000-U; Nikon, Tokyo, Japan), the migration of DNA from the nucleus of each cell was measured with a computer program http://tritekcorp.com using the parameter of the percent of tail DNA [27,28] which was the percentage of DNA in the comet tail (sum of the intensities of pixels in the tail).

### Proliferative inhibition test

The proliferation of cells was determined by a Trypan blue dye exclusion assay as described [7,29]. In brief, cells were treated with the vehicle control (DMSO) or different doses of 4βHWE in media for 24 h. After incubation, cells exposed to 0.2% Trypan blue were counted in a hemocytometer, and cells stained with Trypan blue were excluded. The proliferation rate was calculated according to the percentage of drug-treated cells over vehicle-treated cells. The assay was done in triplicate, and the IC_50 _value was calculated by the slope and intercept according to two concentrations of 4βHWE between the half-maximal proliferative inhibitory concentrations.

### Flow cytometric determination of the cell cycle histogram by PI staining

The cell cycle histogram was determined as described before [29]. In brief, 1 × 10^6 ^cells per 100-mm Petri-dish (at 70%~80% confluency) were treated with the vehicle or 1 μg/mL of 4βHWE for 12 and 24 h. After treatment, cells were collected, washed twice with PBS, and fixed in 70% ethanol overnight. After centrifugation at 700 rpm for 5 min at 4°C, the cell pellet was stained with 10 μg/mL PI (Sigma, St. Louis, MO) and 10 μg/mL RNase A in PBS for 15 min at room temperature in the dark. The analyses were performed with a FACScan flow cytometer (Becton-Dickinson, Mansfield, MA) and Cell-Quest and Modfit software (Becton-Dickinson).

### Assessment of apoptosis

The accumulation of a sub-G_1 _population of H1299 cells was determined by flow cytometry as described above. To further confirm apoptosis in 4βHWE-induced H1299 cells, annexin V and PI double-staining (Pharmingen, San Diego, CA) was performed as described before [30]. In brief, 1 × 10^6 ^cells per 100-mm Petri-dish (at 70%~80% confluency) were treated with vehicle or 51 μg/mL of 4βHWE for 24 h. Subsequently, cells were labeled with annexin V-FITC (10 μg/mL)/PI (10 μg/mL); stained slides were mounted, and analyzed using an inverted fluorescence microscope (TE2000-U; Nikon). Alternatively, the apoptotic ability of treated cells was analyzed using a flow cytometric analysis as described before [31].

### Statistical analysis

All data are presented as the mean ± SD; Student's *t-*test was used to test the mean difference between the two groups.

## Results

### The main pure compound from P. peruviana

**Figure 1:**
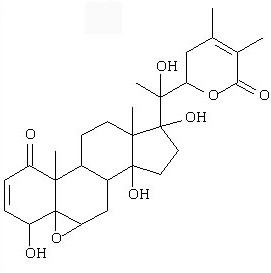
**Chemical structure of 4β-hydroxywithanolide E (4βHWE)**.

The main pure compound isolated from *P. peruviana *was 4βHWE according to the extraction method listed in "Materials and Methods". Accordingly, the total collected plant extract was 10.8 kg, and the yield of 4βHWE was 866.5 mg. The chemical structure of 4βHWE is shown in Figure 1.

### DNA damage determined by the comet assay

**Figure 2:**
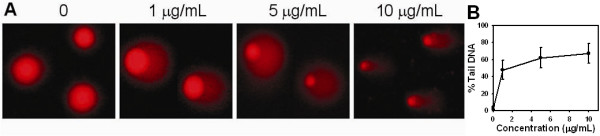
**Comet-NE assay of 4βHWE in H1299 cells**. (A) PI staining for cell controls (DMSO) and 4βHWE-treated cells (1, 5, and 10 μg/mL, 2 h). The circular spots in pink were nuclei; the tails indicated the DNA damage. (B) Average of % tail DNA for 4βHWE-treated cells.

In the comet assay for the H1299 cell line, no "tail" was found in the untreated control (DMSO alone) while the "tail" grew after 4βHWE treatment (1, 5, and 10 μg/mL, 2 h) (Figure 2A). Figure 2B shows that values of the average percent tail DNA for the NE buffer control, DMSO control (0.1%), and 4βHWE treatment (1, 5, and 10 μg/mL, 2 h) were 3.75% ± 2.04%^a^, 3.66% ± 2.83%^a^, 47.89% ± 11.31%^b^, 62.10% ± 11.78%^c^, and 67.38% ± 11.58%^d^, respectively (*p *< 0.05 between different superscript letters). Accordingly, the DNA damage by 4βHWE had a significant dose-dependent effect. These results suggested that DNA damage was involved in the antiproliferation of 4βHWE towards lung cancer cells.

### Proliferative inhibition test

**Figure 3:**
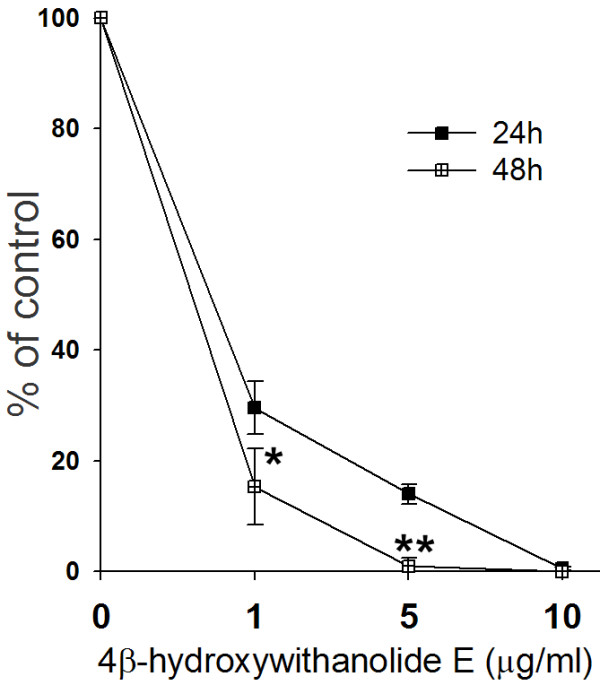
**Proliferative inhibition of 4βHWE on H1299 cells**. Cells were incubated with concentrations of 4βHWE (from 0, 1, 5, and 10 μg/mL) for 24 and 48 h. The proliferation inhibition was determined by Trypan blue assay. The data was mean ± SD, n = 3. The asterisks indicated the statistical significance between drug treatment for 24 h and 48 h at the same concentration according to the Student *t *test (*P *< 0.05* and 0.001**, respectively).

Figure 3 shows that the IC_50 _values of the 4βHWE-treated H1299 cell line for 24 and 48 h were 0.6 and 0.71 μg/mL, respectively. Growth inhibition of H1299 cells by 4βHWE occurred in a dose-responsive and time-dependent manner (*p *< 0.05 and 0.001 for 24 and 48 h, respectively).

### Assessment of cell cycle arrest and apoptosis

**Figure 4:**
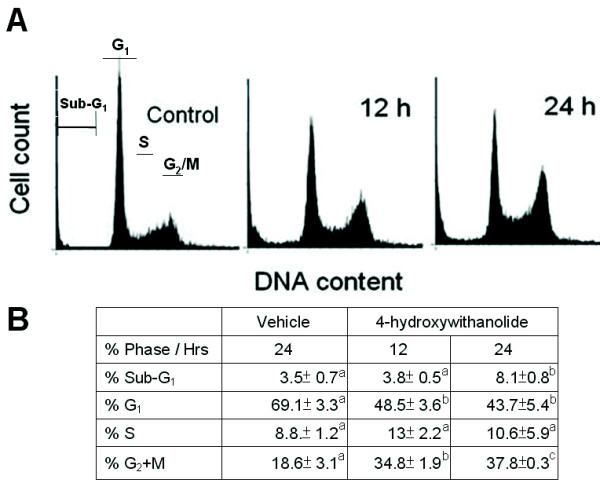
**4βHWE accumulated G_2_/M phase in H129 cells**. (A) The cell cycle distribution for 4βHWE (1 μg/mL for 12 and 24 h) treated H1299 cells and untreated controls. (B) The percentages of the cell cycle phase. The data was mean ± SD, n = 3. Different letter notations indicated the statistical significance between drug treatment and vehicle (*P *< 0.002).

Figure 4A shows the cell cycle distribution of cells treated with the vehicle or 1 μg/mL of 4βHWE for 12 and 24 h; Figure 4B shows their sub-G_1_, G_1_, S, and G_2_/M percentages after drug treatment. It was found that the sub-G_1 _percentages were 3.5% ± 0.7%, 3.8% ± 0.5%, and 8.1% ± 0.8% (*n *= 3) for the vehicle, and 12 and 24 h of treatment with 4βHWE, respectively. G_1 _percentages were dramatically reduced from 69.1% ± 3.3% (vehicle) to 48.5% ± 3.6% and 43.7% ± 5.4% (*n *= 3) with 12 and 24 h of 4βHWE treatment, respectively. G_2_/M percentages were 18.6% ± 3.1%, 34.8% ± 1.9%, and 37.8% ± 0.3% (*n *= 3) for the vehicle or 12 and 24 h of 4βHWE treatment, respectively. Accordingly, moderate sub-G_1 _accumulation and G_2_/M arrest were found in a time-dependent manner for the 4βHWE-treated H1299 cell line. All significant differences between the vehicle and drug treatment are shown in Figure 4.

**Figure 5:**
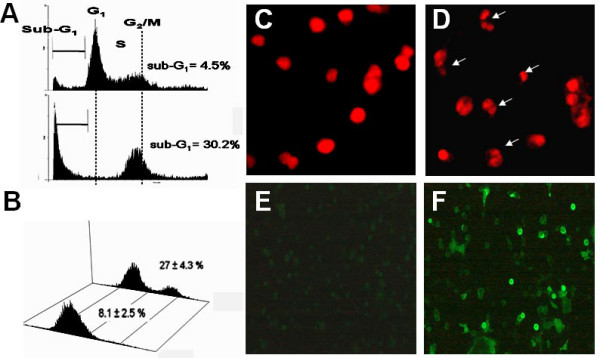
**4βHWE-induced apoptosis in H1299 cells**. Cells were administered with or without 5 μg/mL of 4βHWE for 24 h. (A) The determination of sub-G_1 _DNA content in H1299 cells by flow cytometry. (B) Apoptosis detection by Annexin V flow cytometry. (C-F). The apoptotic phenotype detected by Annexin V/PI double staining was found in (D, F) 4βHWE-treated cells rather than in vehicle (C, E), respectively. Original magnification: × 200.

As shown in Figure 4, cell cycle arrest was not complete, and only moderate accumulation at the sub-G_1 _phase occurred. To perform a confirmatory study, we enhanced the dosage of 4βHWE to validate the cell cycle disturbance (Figure 5A) and apoptotic cell death (Figure 5B). Figure 5A shows that G_2_/M arrest was complete with a higher dose of 5 μg/mL of 4βHWE treatment for 24 h. Meanwhile, percentages of cells with a sub-G_1 _DNA content for the vehicle- and 4βHWE-treated H1299 cell line were 2.5% and 30.2%, respectively, suggesting that sub-G_1 _accumulation was dose-dependent compared to that of Figure 4 (8.1% with drug treatment for 24 h).

Sub-G_1 _accumulation was further confirmed as being due to apoptosis using annexin V/PI double-staining (Figure 5B); i.e., annexin V-positive signals for drug and vehicle treatments were 27.0% ± 4.3% and 8.1% ± 2.5%, respectively. PI staining showed that apoptotic nuclei increased in drug-treated cells (Figure 5D) compared to the vehicle (Figure 5C). Annexin V staining showed strong FITC fluorescence in drug-treated cells (Figure 5F) compared to the vehicle (Figure 5E).

## Discussion

Selective activation of apoptosis is often cited as one of the major goals of cancer chemotherapy. It is currently thought that dysregulated cell cycle proliferation and apoptosis are important factors in the development of cancer [32]. Therefore, it appears that exploiting the apoptotic potential of cancer cells might lead to new therapies that could be less toxic to normal cells due to the physiologically controlled survival pathway [33].

The ethanol extract of *P. peruviana *was reported to possess the lowest IC_50 _value against Hep G_2 _cells but had no cytotoxic effect on normal BALB/C mouse liver cells [19]; however, because these two cell lines are derived from different species, they are unable to provide strong evidence that the extract is a potential chemotherapeutic agent for lung cancer. Using cell lines of the same species, we found that the IC_50 _of 4βHWE at 24 h was 3.75 μg/mL for the HGF-1 (human normal gingival fibroblast) [24] cell line (data not shown) which was higher than the IC_50 _of the H1299 human lung cancer cell line (at 0.71 μg/mL; Figure 3). These results showed that H1299 cancer cells, but not a normal cell line, were sensitive to 4βHWE. However, we cannot exclude the possibility that there are tissue-specific differences between gingival and lung cells. Therefore, further *in vitro *cell cytotoxicity studies using a normal lung cell line, additional different lung cancer cell lines, and an *in vivo *nude mice study are necessary to prove that 4βHWE can inhibit tumor growth without major side effects.

Genomic lesions due to activation of the DNA damage response were detected, which then determined the cell fate, i.e., either cycle arrest or apoptosis (Figures 3, 4). However, both cell cycle interruption and apoptosis analyses are time-consuming and cannot be completed within a short time, e.g., at least 12~24 h of drug treatment is required. In contrast, the comet-NE assay was very sensitive to DNA strand breaks and can rapidly be performed [34], making it possible to determine the extent of DNA damage within only 2 h [26]. Moreover, to confirm the comet assay results, we enhanced the dosage of 4βHWE according to the IC_50 _value; the results of treatment with the IC_50 _and other higher concentrations were similar, and exhibited a dose-responsive effect.

At high concentrations, the ethanol extract of *P. peruviana *induced cell cycle arrest, dose-dependent accumulation of the sub-G_1 _peak, and apoptosis through mitochondrial dysfunction [19]. In this study, the main pure compound, 4βHWE from *P. peruviana*, was used to treat the H1299 lung cancer cell line. The results showed that cell cycle arrest at the G_2_/M phase (Figures 4, 5A), dose-dependent accumulation of a sub-G_1 _peak (Figure 4), and apoptosis were found in drug-treated cells (Figure 5). Furthermore, we found that cell cycle arrest at the G_2_/M phase was mediated by the ATM-NBS1 pathway which was activated by double strand breaks (data not shown).

The conventional alkaline comet assay is sensitive to DNA strand breaks, but is not sensitive to DNA lesions without strand breaks. In contrast, the NE in the comet-NE assay theoretically contains all of the repair enzymes and increases the incision of most kinds of DNA damage. Therefore, the comet-NE assay [34-36] is generally more sensitive than the traditional comet assay. However, different NEs from different cell lines may display differential excision activities for different types of DNA adducts. For example, the NE of NTUB1 cells is sensitive to ultraviolet C-induced DNA damage but is less sensitive to methyl methanesulfonate (MMS) than the NE of BFTC905 cells [34]. Another problem with the comet-NE assay is that details of the types of lesions caused by the DNA damaging agent are not available. To resolve this problem, lesion-specific enzymes [28] can be used to replace the NE which may provide a more-specific and -sensitive way to detect DNA damage. Alternatively, immunodepletion coupled with the comet-NE assay might reveal the specific type of DNA damage [35].

## Conclusions

We demonstrated herein that golden berry-derived 4βHWE had a strong inhibitory effect on the growth of H1299 lung carcinoma cells. This study demonstrated that the DNA damage caused by 4βHWE is the result of a toxicological mechanism. 4βHWE was also demonstrated to have anti-lung cancer activity, along with the potential to produce G_2_/M arrest and apoptosis in time-dependent manners. Our ongoing study further validates the effective cancer-preventive potential of 4βHWE in other cancer cell lines as well as in a xenograft assay. If 4βHWE is indeed proven to exert an effective cancer preventive potential in vivo, golden berry-derived 4βHWE may be a promising ingredient as a cancer-preventive functional food.

## Competing interests

The authors declare that they have no competing interests.

## Authors' contributions

C-YY, C-CC, and H-WC participated in the writing and study design. Y-CW instructed F-RC and Y-HL on extracting the pure compound from the fruit. Y-CH, H-LY, and Y-CL provided instruction for the comet assay and its analysis. M-TT, Y-SC, Y-CC, and Y-CK participated in the comet assay experiments. JY-FC, C-CH, AY-LL, and Z-LG performed the apoptosis analysis. H-WC and Y-CW coordinated and oversaw the study. All authors read and approved the final manuscript.

## Pre-publication history

The pre-publication history for this paper can be accessed here:

http://www.biomedcentral.com/1471-2407/10/46/prepub
